# Collagen-Based Matrices for Osteoconduction: A Preclinical In Vivo Study

**DOI:** 10.3390/biomedicines9020143

**Published:** 2021-02-02

**Authors:** Hiroki Katagiri, Yacine El Tawil, Niklaus P. Lang, Jean-Claude Imber, Anton Sculean, Masako Fujioka-Kobayashi, Nikola Saulacic

**Affiliations:** 1 Department of Cranio-Maxillofacial Surgery, Inselspital, Bern University Hospital, Faculty of Medicine, University of Berne, CH-3010 Berne, Switzerland; katagiri@ngt.ndu.ac.jp (H.K.); y-eltawil@hotmail.com (Y.E.T.); nplang@switzerland.net (N.P.L.); masako.kobayashi@tky.ndu.ac.jp (M.F.-K.); 2 Advanced Research Center, The Nippon Dental University School of Life Dentistry at Niigata, Niigata 951-8580, Japan; 3 Department of Periodontology, School of Dental Medicine, University of Berne, CH-3010 Berne, Switzerland; jc-imber@gmx.net (J.-C.I.); anton.sculean@zmk.unibe.ch (A.S.)

**Keywords:** animal experiment, critical size defect, collagen, hydroxyapatite, micro-CT, histological analysis

## Abstract

The aim of this study was to evaluate the influence of additional hydroxyapatite (HA) in collagen-based matrices (CM) and membrane placement on bone formation in calvarial defects. Critical size defects in the calvaria of 16 New Zealand White Rabbits were randomly treated with CM or mineralized collagen-based matrices (mCM). Half of the sites were covered with a collagen membrane. Animals were euthanized after 12 weeks of healing. The samples were studied by micro-CT and histology. Newly formed lamellar bone was observed in all samples at the periphery of the defect. In the central areas, however, new bone composed of both woven and lamellar bone was embedded in the soft tissue. Samples treated with mCM showed more residual biomaterial and induced more small bony islands in the central areas of the defects than samples with CM. Nevertheless, a complete defect closure was not observed in any of the samples at 12 weeks. Membrane placement resulted in a decrease in bone density and height. Significant differences between the groups were revealed only between CM groups with and without membrane coverage for bone height in the central area of the defect. Neither mineralization of CM nor membrane placement improved the osteogenic capacity in this particular defect. Nevertheless, mineralisation influenced bone density without a membrane placement and bone volume underneath a membrane. CM may be used as a scaffold in bone regeneration procedures, without the need of a membrane coverage. Further preclinical studies are warrant to optimise the potential of mCM.

## 1. Introduction

Bone augmentation procedures are frequently required prior to or simultaneously with implant placement due to insufficient bone volume available. In general, autogenous bone grafts are considered as gold standard for bone augmentation procedures owing to their biocompatibility and their capacity of osteoconduction. The necessity of having to harvest bone grafts is the major shortcoming of this procedure. Moreover, a possible risk of donor site morbidity and other adverse events in neighboring anatomical structures may be encountered [1,2,3,4].

Different bone substitutes are commonly used for bone volume augmentation to avoid autogenous bone grafting. Alloplastic biomaterials, such as calcium phosphates in particular, have been in focus of research due to relative ease in processing and good cell attachment [4,5,6,7]. The most important feature of the biomaterials is the capacity to stabilize the blood clot and prevent flap collapse, maintaining the space needed for the regeneration process. The porosity and pore size of biomaterials are decisive to promote vascularization and osteoconduction [6]. 

Collagen-based materials have been introduced in medicine and dentistry because of their biocompatibility and capability of promoting wound healing [8,9,10,11,12]. Collagen of porcine origin has been frequently used as a barrier membrane in guided tissue (bone) regeneration. The membrane is adapted to the shape of the defect, and the blood clot forming underneath the membrane will be stabilized. Some preclinical studies indicated that a spongy composition of the collagen barrier membranes may even serve as an osteoconductive biomaterial supporting the ingrowth of bony tissue [13]. A recent study showed that Type I collagen mixed with porcine bone substitute (30:70) significantly increased bone formation when compared to porcine bone alone [14]. Fibrillar collagen structure seemed to provide a scaffold for cell ingrowth and regeneration. The potential of collagen as a bone substitute material is, however, unknown.

Different collagen-based biomaterials were introduced into the market for soft tissue augmentation as well, and as an alternative to the free gingival and subepithelial connective tissue grafts [15]. A novel collagen-based tissue matrix (CM) has been shown to possess excellent biocompatibility, favorable soft connective tissue integration and adequate promotion of angiogenesis [11,16,17,18,19,20,21]. Due to its structural configuration (e.g., high porosity and interconnectivity), CM maintains its volume and stabilizes the blood clot [22]. Besides of the restricted epithelium proliferation, a stable blood clot may also maintain a high concentration of autogenous growth factors in the wound. Discrete signs of mineralization within the CM [23] and bone formation at the interface between CM and the underlaying ridge defect were also observed [24].

The present study aimed to assess the influence of the CM on healing of critical-size bone defects in rabbits calvaria using the principle of guided bone regeneration (GBR). The impact of hydroxyapatite (HA) on bone formation and graft resorption was determined by means of micro-computed tomography (micro-CT) and histology. 

## 2. Materials and Methods

### 2.1. Biomaterials

**Figure 1:**
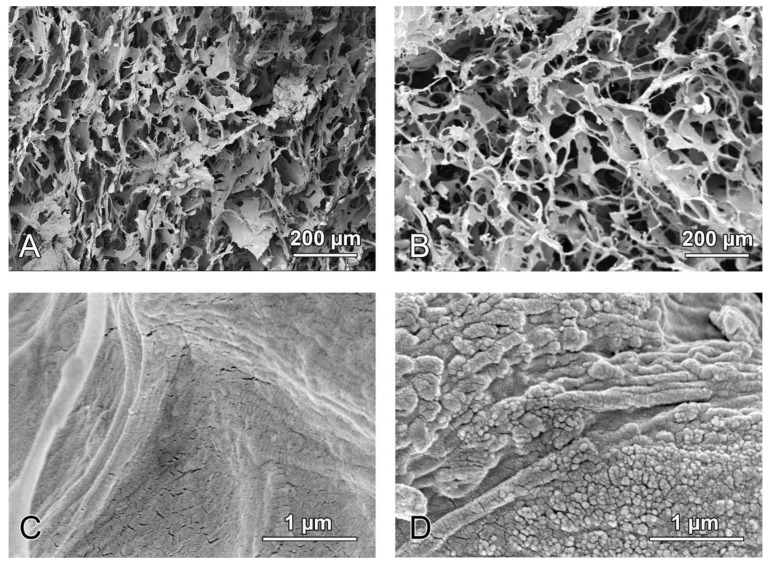
SEM images of the CM, (**A**,**C**) and mCM (**B**,**D**). Both CM and mCM showed relatively smooth surface at low magnification because of cross-linking of collagen. The mCM included additional hydroxyapatite particles on the collagen surfaces (**D**).

**Figure 2:**
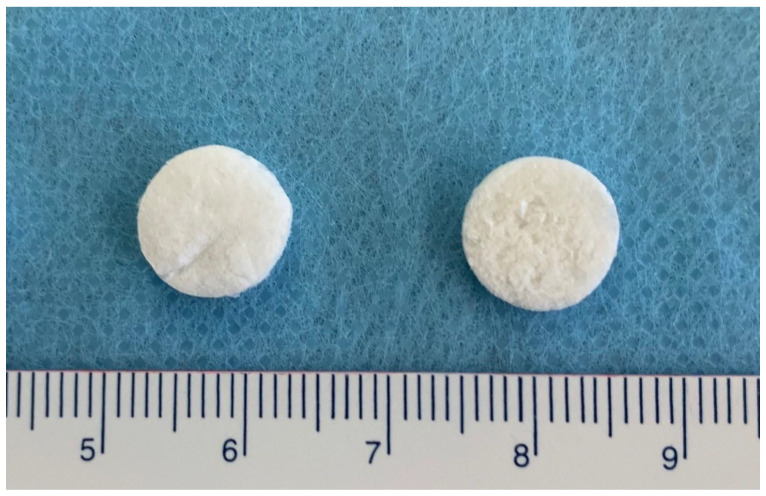
CM (left) and mCM (right) pre-shaped for implantation (φ= 10 mm, thickness = 2 mm).

A CM (Fibro-Gide^®^, Geistlich Pharma AG, Wolhusen, Switzerland) is a cross-linked, porcine-origin collagen sponge, made of reconstituted collagen Type I and III. It is a porous, resorbable and volume-stable biomaterial, specifically designed for soft-tissue augmentation. Overall, two collagen tissues were isolated, processed, milled, and mixed at a 3.5–4.5:0.5–1.5 ratio. After freeze-drying, the resulting composite was treated with a crosslinking agent, washed with water, freeze-dried again, and finally sterilized by gamma irradiation. The detailed production process is proprietary. The final product was composed of 60–96% (w/w) porcine collagen Type I and III and 4–40% elastin. Furthermore, the average pore diameter was 92 µm, and the volume porosity was 93% with interconnected pores. Chemical cross-linking was used to increase the stiffness of the scaffold. Mineralized CM (mCM) is a 15–20% HA shaped composite of CM. The scanning electron microscope (SEM) images of CM and mCM (DSM 982 Gemini digital field emission SEM, Zeiss, Oberkochen, Germany) were shown in Figure 1. Both biomaterials were separately packed before surgery. Before implantation, CM and mCM were pre-shaped in a 10-mm diameter discs and sliced with a scalpel to the thickness of 2 mm (Figure 2).

### 2.2. Animals

We used 16 New Zealand white female rabbits (3.3–3.8 kg), approximately 16 weeks of age. During the acclimatization period and throughout the experiment, the animals were housed in the Central Animal Care Facility at the University of Berne (temperature 19–21 °C, humidity 45% ± 10%, a light–dark cycle of 12:12 h). The animals were housed without excessive or disturbing noises and fed with a standard diet and water ad libitum. The study considered National Centre for the Replacement Refinement & Reduction of Animals in Research (NC3Rs) and is reported according to the Animal Research: Reporting of In Vivo Experiments (ARRIVE guidelines) for preclinical in vivo studies. The study was submitted to and approved by the Committee for Animal Research, Canton of Berne, Switzerland (Nr: BE 89/17). 

### 2.3. Anesthetic Procedure

Before surgery, the animals were pre-medicated with methadone (0.3 mg/kg), dexmedetomidine (100 g/kg) mixed with ketamine 15 mg/kg (Narketan^®^, Vetoquinol AG, Berne, Switzerland), s.c. in the neck area. The animals were left undisturbed for 5 to 10 min. After reaching an appropriate depth of sedation, the eyes were lubricated (Bepanthen^®^ Augen- und Nasensalbe, Bayer Vital GmbH, Leverkusen, Germany) and pure oxygen administered by a facemask. An intravenous (iv.) catheter was inserted in one of the marginal auricular veins. After clipping and disinfection, ropivacaine 0.75% was administered on the surgery site. General anesthesia was maintained with isoflurane (Forene^®^, Abbvie AG, Baar, Switzerland) vaporized in pure oxygen through a Jackson Rees modified T-piece breathing system targeting a maximal Et Iso of 1–1.3%.

Perioperative antimicrobial prophylaxis consisted of procaine penicillin 150,000 IU/mL + benzathine penicillin 150,000 IU/mL (Duplocillin^®^, MSD Animal Health, Luzern, Switzerland) 0.01 mL/kg s.c. before surgery. 

### 2.4. Surgical and Postoperative Procedures

**Figure 3:**
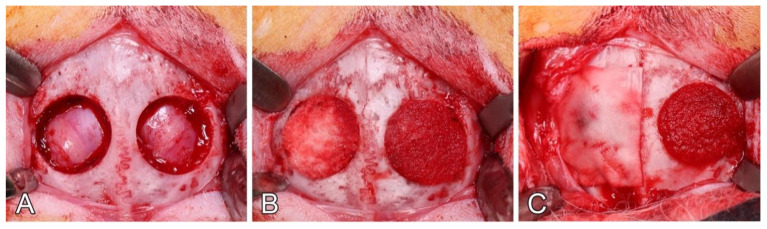
Intrasurgical view of the exposed rabbit calvaria. (**A**); Two 10 mm diameter defects were created in the parietal bone with a trephine drill. (**B**); Allocated materials were implanted in the defects: left defect, CM; right defect, mCM. (**C**); Left defect is covered with a collagen barrier membrane (12 × 12.5 mm).

The skin was incised from the nasal bone to the mid-sagittal crest, and the periosteum was elevated to expose the parietal bone. The two 10-mm diameter calvarial bone defects were prepared with a trephine under copious irrigation with sterile saline. Maximal care was taken to avoid injury to the dura mater (Figure 3a). Both biomaterials were implanted in the critical-size defects without excessive pressure (Figure 3b). Once the biomaterials were soaked with the blood, a non-cross-linked, porcine collagen membrane (Bio-Gide^®^, Geistlich Pharma AG, Wolhusen, Switzerland) was used to cover the half of the sites treated with CM or mCM (Figure 3c). Correspondingly, the animals were divided into 4 groups: CM alone, mCM alone, CM covered with membrane (CM/M) and mCM covered with membrane (mCM/M). Allocation of the applied treatment modalities was randomized according to the systematic random protocol (www.randomization.com). Defect areas were closed in two layers with interrupted sutures using 4–0 Vicryl^®^ and 4–0 Monocryl^®^ sutures (Ethicon, Somerville, NJ, USA), respectively. Wound surfaces were sealed with a spray film dressing (OPSITE^®^ SPRAY, Smith and Nephew, London, UK).

The rabbits were left to recover under infrared lights and administration of oxygen following surgery. Postoperative analgesia consisted of meloxicam (Metacam^®^, Boehringer Ingelheim, Ingelheim, Germany) 0.5 mg/kg i.v. administered after surgery and repeated once daily for 4 days. Regular monitoring included assessment of water and food consumption and pain at regular intervals (composite pain scale and grimace scale). If indicated, Buprenorphine (Temgesic^®^, Rechitt Benckiser, Wallisellen, Switzerland) was administered at 20 g/kg s.c. every 8 h during 3 days.

### 2.5. Bone Labeling

For the labeling of bone formation and remodeling, the rabbits were injected with Calcein 10 mg/kg i.p., (Sigma, St. Louis, MO, USA) immediately after the surgery, Tetracycline 25 mg/kg s.c. (Sigma, St. Louis, MO, USA) at 6 weeks after surgery and Alizarin Red 30 mg/kg s.c. (Alizarin red S, Sigma, St. Louis, MO, USA) at 3 days before euthanasia. 

### 2.6. Sacrifice

Only one rabbit had to be euthanized on the first day after surgery because of neurological symptoms. Thus, 15 rabbits were sacrificed after the healing period of 12 weeks. Rabbits were euthanised with an overdose of pentobarbital 120 mg/kg i.v. (Streuli Pharma AG, Uznach, Switzerland) following the premedication with ketamine 65 mg/kg and xylazine 4 mg/kg s.c. in the neck area. After sacrifice, the harvested calvarial bone including implanted biomaterials were processed for micro-CT and histological analysis. 

### 2.7. Micro-CT Analysis

Following harvesting, the corrected calvaria bone were fixed in 10% neutral formalin for 7 days at room temperature then replaced in 70% ethanol at 4 °C. The specimens were then subjected to micro-CT scans using a desktop cone beam scanner (micro-CT 40, ScancoMedical AG, Brüttisellen, Switzerland). The X-ray source was set at 70 kV with 114 μA. An isotropic voxel size of 18 μm showed an image matrix of 2048 Å~ 2048 pixels. The micro-CT images were then analyzed and reconstructed by using 3D structural analysis software (Amira, Visualization Sciences Group, Düsseldorf, Germany). The volume of interest (VOI) was a 10-mm diameter, full thickness cylinders, selected corresponding to the dimensions of the defect sites. 2D parameters included defect closure measured on the horizontal plane (DC, relative % to whole surface at the defect site) and bone height (BH) measured on the sagittal plane, at the middle 5 mm (BH_M) and at the lateral part of the defect (BH_L). Bone volume (BV, mm^3^), BV fraction (BV/TV, ratio of the segmented BV to the total VOI) and relative BD (BD, relative % to initial bone at the defect site) were calculated. The micro-CT data of the harvested bone disc was used as reference threshold values for the BD analyses of tested groups.

### 2.8. Histological Processing and Histomorphometric Analysis

All specimens were trimmed, dehydrated in ascending concentrations of ethanol, and embedded in methyl methacrylate without decalcification. The embedded tissue blocks were cut sagittally at the middle of the defects into approximately 800 μm thick ground sections using a slow-speed diamond saw (VC50; LECO, St. Joseph, MI, USA). After mounting on acrylic glass slabs, the sections were ground and polished to a final thickness of 200 μm (Knuth Rotor-3; Struers, Ballerup, Denmark). The labeled bone was digitally photographed under a fluorescent microscope (Nikon Eclipse E800; Nikon, Tokyo, Japan). The sections were stained with toluidine blue combined with fuchsin and the images photographed under a digital microscope (VHX-6000, Keyence, Japan). Morphometric analysis was performed by a graphic software (Photoshop CC; Adobe, San Jose, CA, USA) using the corresponding 10-mm initial defect area as the region of interest (ROI). Linear parameters included horizontal defect closure (HDC, %) and the new bone height (BH, mm), measured in the middle (BH-M) and in the lateral parts of the defect (BH-L). New bone area (NBA), bone marrow area (BMA), residual material area (RMA) and connective tissue area (CTA) were measured as a relative % to the total augmentation area.

### 2.9. Statistical Analysis

The means and all plots of values were represented for all quantitative data. The statistical analysis was done by one-way analysis of variance (ANOVA) with Tukey test using a statistical program (GraphPad Prism 7.0 software; GraphPad Software, Inc., La Jolla, CA, USA). The level of significance was set at α = 0.05. 

## 3. Results

At harvesting, no signs of inflammation, infection, wound dehiscence or exposure of the surgical sites were observed. However, one defect had to be excluded from the analysis due to the damage imposed on the dura mater. A total of 29 defects (*n* = 29) were analyzed. A sample size per healing period consisted of eight defects (*n* = 8) for the CM group, and seven defects (*n* = 7) for the mCM, CM/M, and mCM/M groups, respectively.

### 3.1. Micro-CT Analyses

**Figure 4:**
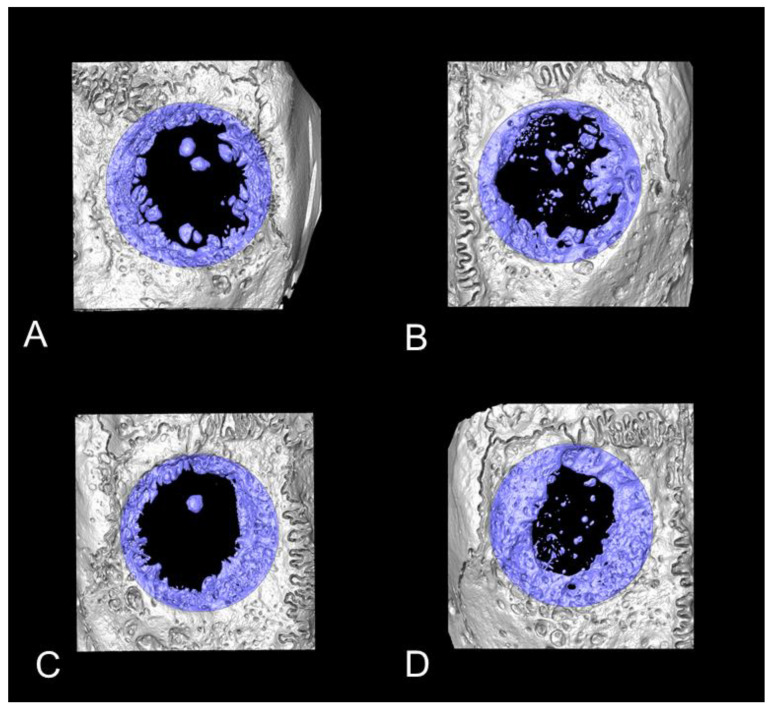
The micro-CT images of each group (top view). (**A**); CM supported bone formation from defect boarder. (**B**); mCM induced not only bone ingrowth from defect border, but also lots of small island-like newly formed bone in the middle of defect. (**C**); CM/M is showing similar bone formation from defect border as CM group. However, collagen barrier membrane did not promote additional bone formation. (**D**) mCM/M is demonstrating greater bone formation from defect border compared to mCM.

**Figure 5:**
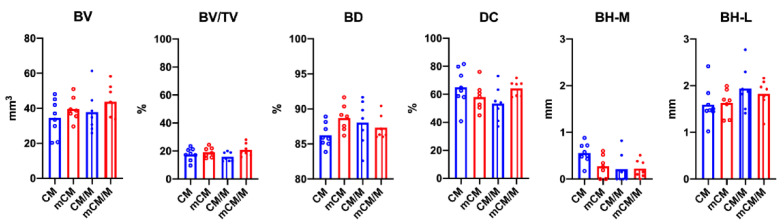
The results of micro-CT morphometry at 12 weeks post-surgery. Any of the groups did not show statistically significant differences, but mCM/M group showed greater bone formation from the defect boarders when compared to mCM alone group.

None of specimens led to the complete closure of the bony defect during the observation period. All groups of biomaterials supported bone formation from the defect boarders (Figure 4). The mineral components of mCM could not be detected by micro-CT. CM/M supported bone formation from the defect boarders as well as CM alone, but the membrane did not promote additional bone proliferation. Interestingly, mCM/M resulted in greater bone formation from the defect boarders when compared to mCM alone. Nevertheless, micro-CT analyses showed that mCM/M tended to induce greatest bone formation among all groups. Yet, none of these differences reached statistical significance (Figure 5). 

Mineralization was found to positively contribute to the BD (mCM, 3971.44 ± 85.21 vs. CM, 3860.78 ± 75.83; *p* = 0.021) and negatively for BH in the middle of the section (BH-M) (mCM, 0.27 ± 0.23 vs. CM, 0.55 ± 0.21; *p* = 0.30) without membrane placement, and positively for BV/TV underneath the membrane (mCM/M, 20.86 ± 4.55 vs. CM/M, 15.84 ± 3.28; *p* = 0.37). Membrane placement significantly decreased BH-M for CM (CM, 0.55 ± 021 vs. CM/M 0.21 ± 0.32, *p* = 0.38) and BD for mCM (mCM, 4002.47 ± 76.47 vs. mCM/M, 3909.22 ± 77.29; *p* = 0.43). When all groups were pooled for membrane placement, mean BH-M was higher for samples without (CM and mCM, 0.55 ± 0.21) than with membrane (CM/M and mCM/M, 0.27 ± 0.23; *p* = 0.30).

### 3.2. Histological Analyses

**Figure 6:**
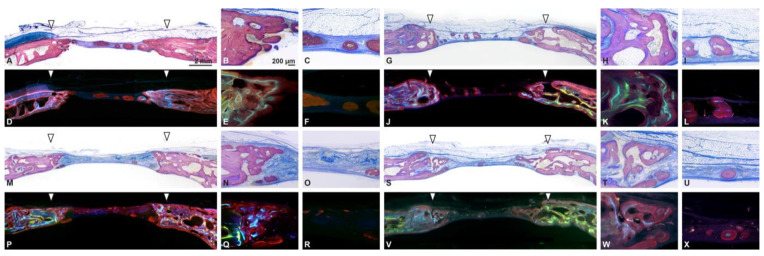
Histological view of the defect (sagittal plane) in CM (**A**–**F**), mCM (**G**–**L**), CM/M (**M**–**R**) and mCM/M groups (**S**–**X**). Overviews (**A**,**M**,**G**,**S**) and magnified views (**B**,**C**,**N**,**O**,**H**,**I**,**T**,**U**) stained with Toluidine blue and Fuchsin. Overviews (**D**,**P**,**J**,**V**) and magnified views (**E**,**F**,**Q**,**R**,**K**,**L**,**W**,**X**) of the merged bone labeling (0w; labeled with calcein in green, 6 w; tetracycline, light blue, 12 w; alizarin, red).

Similar levels of bone formation were observed among the groups. All groups demonstrated new bone formation starting from the edges of the bony defects (Figure 6). The thickness in the lateral bone of the ROI was well maintained in all groups. Both groups without membrane coverage (CM and mCM) showed newly formed bone within the biomaterial in the centre of the defects. Residual collagen fibers were observed in all samples (Supplementary Figures S1–S4). The remnants of collagen materials were, in general, surrounded by multinucleated giant cells (MNGCs) and isolated from surrounding connective tissue. This cellular reaction was accompanied with a lymphocytic infiltration. In comparison to the CM, mCM induced island-like osteoid tissue as hydroxyapatite particles appeared to be a “nucleus” of bone formation (Supplementary Figures S2 and S4). The HA particles were covered with osteoid, while the new bone was surrounded by MNGCs accompanying by a lymphocytic infiltration. Because of the HA particles, mCM and mCM/M showed the remnants of the biomaterial more frequently. The frequency of the residual material observed on the sections of CM, mCM, CM/M and mCM/M was 37.5, 57.1, 28.6, and 71.4%, respectively. Additional membrane coverage slightly impaired bone formation, especially in the center of the defect. Both groups without membrane coverage seemed to better maintain the connective tissue height. 

**Figure 7:**
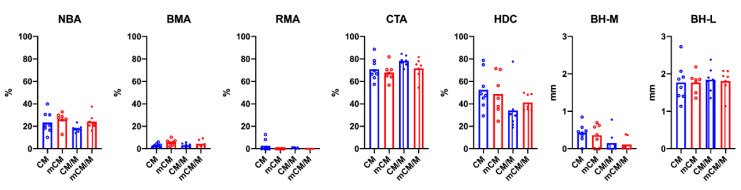
The comparison of histomorphometric results. The mCM group showed highest mean values of NBA and BMA. No significant differences were observed among the groups.

The mCM group showed highest mean values of NBA and BMA (Figure 7). Nevertheless, no significant differences were observed for any area parameters between the four groups. Mineralization of CM significantly increased BMA without membrane placement (mCM 5.74 ± 2.73 vs. CM 3.01 ± 1.62, *p* = 0.045) and reduced RMA (CM/M 0.71 ± 0.68 vs. mCM/M 0.00 ± 0.00, *p* = 0.032) underneath the membranes. Highest mean HDC and BH-M were observed for CM, without reaching statistical significance for any of the linear parameters between the groups. When all groups were pooled for membrane placement, mean BH-M was significantly higher for samples gathered without (CM and mCM, 0.39 ± 0.25) than with membranes (CM/M and mCM/M, 0.13 ± 0.23; *p =* 0.009). 

## 4. Discussion

The aim of this study was to investigate the possibility to enhance the osteogenic potential of CM by mineralization under the principle of GBR. Coagulum stability and space provision are considered the most important prerequisite for bone regeneration. The tested volume-stable, cross-linked CM did not only stabilize the blood clot, but was, additionally, acting as a scaffold for progenitor cell invasion [11]. The analysis revealed some possible changes in biomaterial resorption and bone formation. The overall impact of the mineralization and membrane placement in the present model was, however, modest. Several issues have to be considered regarding these findings. 

The standardized, membrane-protected defects in the calvaria of the rabbit are highly suitable to detect bone formation in the presence of bone substitutes. Biomaterials act as a scaffold to support or encourage the innate regenerative capabilities of the host’s own cells to form bone. The present results correspond to previous findings in a study on soft tissue regeneration where bone was formed without distinctive borders to the CM and surrounding encapsulation [23]. The cell homing concept of biomaterials in the form of scaffolds could thus be a possible explanation for the observed CM integration in the new tissues. This, in turn, supports the bone formation observed from the periphery to the defect. In the present study, immunohistochemistry was not performed. Hence, the origin of the newly formed soft tissue surrounding bone cannot be determined with certainty. 

In contrast to the model where CMs were implanted over the pristine bone for soft tissue regeneration [11,23,25,26,27], CMs in the present study were placed into standardized defects in the calvaria for the assessment of bone formation. Critical-size defects were used as the most well-established model for studying bone formation [28,29,30]. Depending on the size of the calvaria defects, more rapid or slower bone closure has to be expected [31,32]. The 10 mm defect size that ultimately would not spontaneously heal was rather challenging. 

The presence of MNGC around remnants of collagen fibers and HA particles with lymphocytic infiltration, however, suggests an extended inflammation (M1 macrophages). MNGC were found at the interface with the surrounding soft tissue expressing cathepsin K, tartrate-resistant acid phosphatase (TRAP) and CD86 that play a role in the degradation of CM [25]. Cathepsin K and TRAP seem to be involved in the degradation of collagen, whereas elastin fibers detected at day 15 appeared to be released from the collagen-elastin scaffold [25]. MNGCs in the present study were detected throughout the defect, corresponding to the findings of Thoma et al. [23], and showing signs of inflammation during the first 2 months of healing. 

The inflammatory phase was followed by rapid cell proliferation and influx of blood vessels and mesenchymal cells towards the center of the CM in another recent study [11]. The presence of bone formation around HA particles in the present study suggests a shift from M1 to tissue regeneration (M2 macrophages). Besides the biomaterial degradation, macrophages are known to regulate the vascularization [26], and the rapid synthesis of collagen fibrils [27]. The properties of the biomaterial may further influence the shift from M1 to M2; the micro-architecture and structure of CM may facilitate the nutrient and oxygen diffusion. The overall impact of CM mineralization to support bone formation in the defects of the present study, however, was weak. Both BV and BV/TV were higher for mCM than for CM. 

Membrane placement had a negative impact especially on BH-M, as demonstrated by both micro-CT and histologically. By protecting the defects from an ingrowth of soft tissue, the use of a resorbable, semi-permeable membrane may promote the process of bone formation. However, in the present study, this possible mechanism for the bone proliferation was not detectable. The reason may have been a collapse into the bone defect [33]. Moreover, the biomaterial used in the present study may have led to a local inflammatory response with oedema, hereby exercising pressure. 

Besides, the stabilization of the bone volume, the CM is accompanied by an increase in volume of approximately 3–12% by hydration from blood [34]. The biomaterial was previously shaped according to the dimensions of the defect and placed without pressure, as squeezing could cause an additional swelling of up to 25% [34]. Furthermore, a volume increase at 1-month post implantation was demonstrated in vivo [23,24]. Taken together, the use of membranes to cover CM does not seem to be justified. 

The knowledge about the later healing events showing the dynamics of cell migration and invasion into the pores of the CM is still missing. An appropriate rate of bone graft degradation corresponding to the degree of bone formation should be provided, as it is demonstrated to be decisive for bone regeneration [35]. The inflammatory reaction to cross-linked collagen biomaterial may delay the tissue integration [36], but the sequence of healing and tissue regeneration at 6 months showed an absence of inflammation [23], remodeling and marked degradation of CM [23,37]. Including longer healing periods, where more advanced bone formation could be expected, should leave sufficient room for a grafting material to reveal its possible superiority in terms of forming new bone.

In the present study, a sham operation or negative control was not performed. It may be speculated that bone formation, to some extent, will succeed even in a negative control without any treatment. Data available in the literature for empty defects with the same anatomies and healing periods in the same species revealed the range of BV/TV between 11 and 36% [38,39]. These findings, thus, were lower [38] or higher [39] when compared to the present results. Nonetheless, the CM of the present study had been designed for soft tissue regeneration and hence, is not supposed to improve the outcomes when compared to an empty control. The primary intention of the present study was to assess if the osteogenic capacity of CMs could be enhanced by mineralization rather than by the biomaterial per se. 

The results of the present study have, for the first time, provided evidence for the potential of this novel CM to facilitate bone regeneration, thus warranting further preclinical testing. Our preliminary study confirmed that CM might have a potential as a scaffold, and that mCM could be an innovative principle for future bone augmentation. The present experiment was designed to provide the scientific basis for future studies. In the present study, the statistical power was sufficient to reveal tendencies, but not significant differences. The sample size was intentionally minimized to decrease the total number of animals used. Furthermore, the size of the rabbit calvaria limits the number of the bone defects per animal. Future research shall be undertaken including a less demanding model for bone regeneration, i.e., non-critical size, self-contained bone defects, without membrane placement. Consequently, the number of defects per group could be increased using the same number of animals. To elucidate the effects of mineralization on the osteogenic potential of CMs, logical aspects of study should include the application of CMs with various percentages of HA. 

## 5. Conclusions

Within the limits of this study, both CMs showed biocompatibility and osteoconduction. Nevertheless, no statistically significant differences were observed for any parameter between four modalities. The impact of mineralization of CMs on bone formation and graft resorption in standardized, critical-size defects in the calvaria of rabbits was modest. Mineralization of CMs significantly increased bone density and bone marrow area in the absence of membrane placement. Moreover, mineralization of CMs increased the fraction of new bone volume underneath a membrane. Furthermore, membrane placement decreased new bone height in the middle of the defects. The present findings should provide evidence for the design of future studies.

## Data Availability

Not applicable.

## Supplementary Materials

The following are available online at https://www.mdpi.com/2227-9059/9/2/143/s1, Figure S1: Observation of residual materials in CM group at 12 weeks after surgery.; Figure S2: Observation of residual materials in mCM group at 12 weeks after surgery; Figure S3: Observation of residual materials in CM/M group at 12 weeks after surgery, Figure S4: Observation of residual materials in mCM/M group at 12 weeks after surgery.
