# Supplementary material for: Collagen-Based Matrices for Osteoconduction: A Preclinical In Vivo Study
Source: Biomedicines. 2021 Feb 2;9(2):143. doi: 10.3390/biomedicines9020143 (PMC7913003; doi:10.3390/biomedicines9020143)
Supplement: Supplementary file 1 [file biomedicines-09-00143-s001.pdf]

Supplementary material

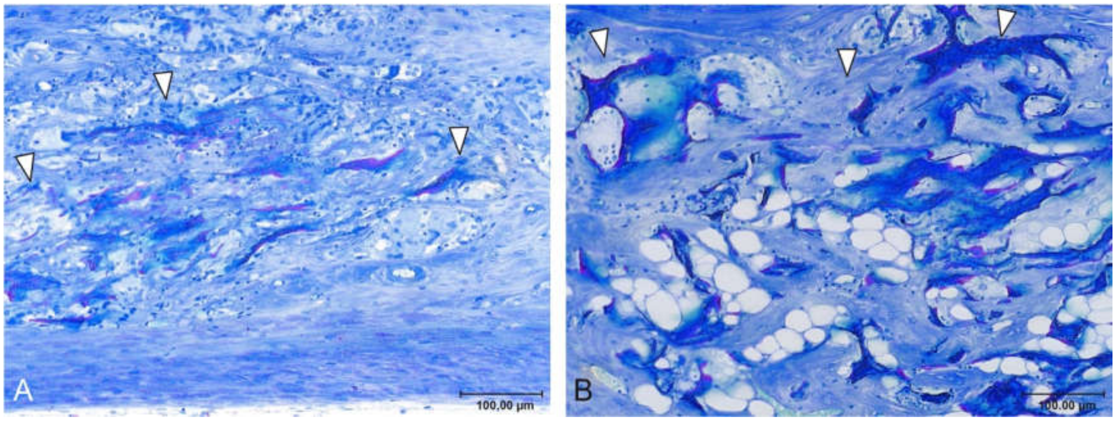

Suppl Figure 1. Observation of residual materials in CM group at 12 weeks after surgery. (A), (B); Residual collagen fibers surrounded by MNGCs (indicated with white arrowheads, bar = 100μm)

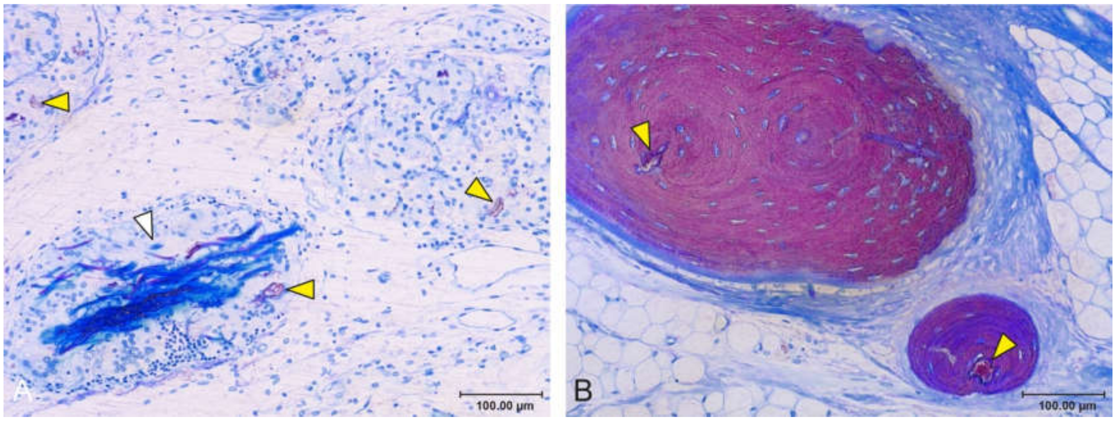

Suppl Figure 2. Observation of residual materials in mCM group at 12 weeks after surgery. (A); Residual collagen fibers (white arrowheads) and, hydroxyapatite particles (yellow arrowheads). (B); residual hydroxyapatite particles surrounded by new bone (yellow arrowheads).

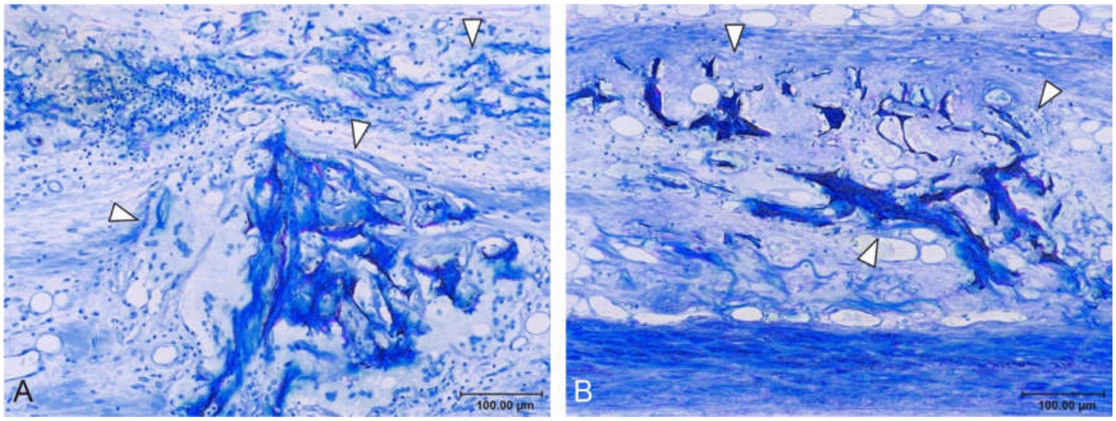

Suppl Figure 3. Observation of residual materials in CM/M group at 12 weeks after surgery. (A); Residual collagen fibers surrounded by MNGCs accompanying the lymphocytic infiltration (white arrow heads). (B); Residual collagen fibers surrounded by dense fibrous connective tissue and few MNGCs.

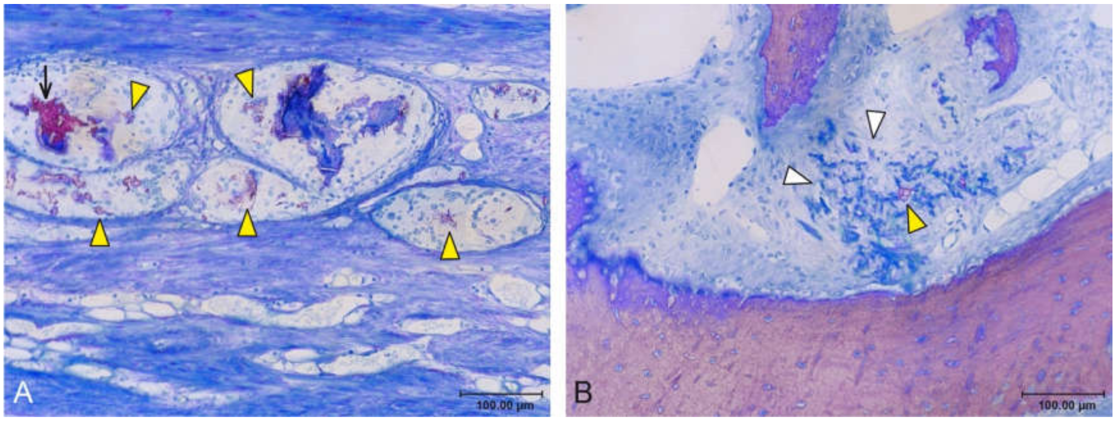

Suppl Figure 4. Observation of residual materials in mCM/M group at 12 weeks after surgery. (A), (B); Residual collagen fibers (white arrowheads), and hydroxyapatite particles (yellow arrowheads), and osteoid was sometimes observed near hydroxyapatite (arrow).
